# Challenges and opportunities of diagnostic markers of Alzheimer's disease based on structural magnetic resonance imaging

**DOI:** 10.1002/brb3.2925

**Published:** 2023-02-16

**Authors:** Xiang Hu, Maria Meier, Jens Pruessner

**Affiliations:** ^1^ Department of Psychology University of Konstanz Konstanz Germany

**Keywords:** Alzheimer's disease (AD), biomarker, hippocampus, structural magnetic resonance imaging (MRI), ventricle

## Abstract

**Objectives:**

This article aimed to carry out a narrative literature review of early diagnostic markers of Alzheimer's disease (AD) based on both micro and macro levels of pathology, indicating the shortcomings of current biomarkers and proposing a novel biomarker of structural integrity that associates the hippocampus and adjacent ventricle together. This could help to reduce the influence of individual variety and improve the accuracy and validity of structural biomarker.

**Methods:**

This review was based on presenting comprehensive background of early diagnostic markers of AD. We have compiled those markers into micro level and macro level, and discussed the advantages and disadvantages of them. Eventually the ratio of gray matter volume to ventricle volume was put forward.

**Results:**

The costly methodologies and related high patient burden of “micro” biomarkers (cerebrospinal fluid biomarkers) hinder the implementation in routine clinical examination. In terms of “macro” biomarkers‐ hippocampal volume (HV), there is a large variation of it among population, which undermines its validity Considering the gray matter atrophies while the adjacent ventricular volume enlarges, we assume the hippocampal to ventricle ratio (HVR) is a more reliable marker than HV alone the emerging evidence showed hippocampal to ventricle ratio predicts memory functions better than HV alone in elderly sample.

**Conclusions:**

The ratio between gray matter structures and adjacent ventricular volumes counts as a promising superior diagnostic marker of early neurodegeneration.

## Graphical abstract

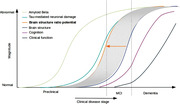

## 1 INTRODUCTION

Dementia encompasses various diseases that are associated with a progressive and irreversible decline in cognitive capacity. According to the World Health Organization (WHO), around 50 million people worldwide are presently being affected by dementia, with 10 million new cases being reported every year (WHO, 2019). The total number of people suffering from dementia is projected to be 82 million in 2030 and 152 million in 2050. In 2015, the total global societal cost of dementia was estimated to be US$818 billion, accounting for 1.1% of the global gross domestic product. Overall, dementia exerts a huge burden on the public health system, with the situation turning for the worse as the aging population is growing, if no effective treatments are identified.

There are different forms of dementia including vascular dementia, dementia with Lewy bodies, frontotemporal dementia, and Alzheimer disease (AD). AD is the most common form of dementia contributing to 60%−70% of the cases. Typically, diagnosis of AD is based on its clinical manifestation, encompassing a range of cognitive symptoms with mild but progressive anterograde amnesia being the most typical early sign. Yet, there is a long presymptomatic phase, likely lasting decades, between the beginning of neuronal degeneration in the brain and the first clinical symptoms (Braak & Braak, 1991; DeKosky & Marek, 2003; Delacourte et al., 1999; Jack et al., 2013). Although the progress of AD is irreversible until an effective treatment can be identified, studies have shown that pharmacological and behavioral intervention at the preclinical stage can prolong the time until clinical symptoms appear. This is important because AD is a disease that occurs in old age. An argument can be made that instead of finding a treatment for AD, it is sufficient to postpone the onset of the disease past the natural life expectancy of the individual. Even if that will not immediately be possible, every year that the onset of the disease can be pushed back will be 1 year where subjects can enjoy living independently with a high quality of life, without any burden on healthcare system and caregivers. Among the behavioral interventions are regular exercise (Schuit et al., 2001; van Gelder et al., 2004), quitting smoking (Anstey et al., 2007; Barnes & Yaffe, 2011), not drinking alcohol excessively (Koch et al., 2019), keeping a healthy weight (Horie et al., 2016), healthy diet (Knight et al., 2016; Shannon et al., 2019), normotension (de Heus et al., 2019; Qiu et al., 2005), and normal blood sugar levels (Zheng et al., 2018).

The presymptomatic period is the ideal stage for both pharmacological and behavioral interventions, for those at risk of developing AD. While most of the behavioral interventions are sound recommendations for anybody, the pharmacological interventions are costly and carry potential side effects. As such, these interventions should target predominantly those at risk. This creates the need to find reliable tools to identify persons, in which the disease has begun to take hold in the brain, but who are not yet symptomatic. Currently, a myriad of studies have tested the reliability and validity of various biomarkers in predicting the insidious degeneration in the brain at an early, presymptomatic stage of AD (Aschenbrenner et al., 2018; Blennow & Zetterberg, 2018; Davis et al., 2018; Dubois et al., 2014; Teipel et al., 2015; Vemuri et al., 2009). In this context, several biomarkers have been shown to be sensitive for the prediction of AD, and there exist several reviews that give an overview of these biomarkers (McGhee et al., 2014; Olsson et al., 2016; Risacher & Saykin, 2013; Shui et al., 2018). Depending on complexity and cost, these markers vary in their ease to implement them in daily clinical practice.

Here, we summarize early diagnostic markers of AD with a focus on ease of use and cost in clinical practice. We end by putting forward that a ratio between brain region volume and its directly adjacent space, which would occupy the region in case of atrophy, is a more informative marker compared to absolute brain region magnitude alone. We summarize promising first evidence from the hippocampal‐to‐ventricle ratio (HVR) and elaborate on its principle and possible advantages compared to other markers.

## 2 EARLY DIAGNOSTIC MARKERS OF AD BASED ON PATHOLOGY ON THE MICRO LEVEL

Neuropathological changes occur many years before clinical manifestations of AD (Braak & Braak, 1991; DeKosky & Marek, 2003; Delacourte et al., 1999; Jack et al., 2013). Already at presymptomatic stages of AD, pathological neurofibrillary tangles (NFTs) composed of phosphorylated tau protein accumulate in brain cells. Further, different isoforms (Qiu et al., 2015) of amyloid deposits of amyloid‐β (Aβ) peptides accumulate in the extracellular space. Those brain proteins are secreted to the cerebrospinal fluid (CSF) (Seubert et al., 1992; Wolozin & Davies, 1987), where they can be detected in CSF (Blennow et al., 2010).

On the basis of these pathological changes on the micro perspective, a variety of biomarkers have been developed to detect beginning AD. First, there is tau protein, which is a group of highly soluble protein isoforms that stabilize microtubules in axons. When looking at NFTs, total tau (T‐tau) and phosphorylated tau (P‐tau) are usually assessed. Hyperphosphorylation of tau proteins can cause NFTs that contribute to the pathology of AD (Iqbal et al., 2005). CSF T‐tau is associated with acute neuronal injuries, reflecting current neurodegeneration burden from days to weeks (Blennow & Hampel, 2003; Zetterberg et al., 2006), while P‐tau does not change with acute brain injury (Zetterberg et al., 2006). High P‐tau levels reflect the chronic phosphorylation degree of tau that is only found in AD but not in other neurodegenerative disorders (Blennow & Zetterberg, 2018). In view of this, CSF P‐tau represents a more specific biomarker for AD as compared to CSF T‐tau, although the two are often highly correlated.

Next, there is amyloid beta (Aβ), which represents peptides of amino acids that mainly constitute amyloid plaques found in the brain of AD patients (Hamley, 2012). When focusing on Aβ, total Aβ peptide accumulation does not seem to be a convincing marker to detect AD. Total Aβ CSF concentration is usually indexed by the most represented isoform—Aβ40 (Aβ with 40 residues; other isoforms are named analogously) (Hansson et al., 2019). Aβ40 levels have, however, been shown to not be specific enough to differentiate between AD and control groups (Shoji et al., 1998). In contrast, focusing on other isoforms of Aβ seems to be more promising. For example, decreased Aβ42 harbors a much stronger potential to reveal imminent AD, since it likely reflects the deposition of amyloid plaque in the brain that gradually leads to brain atrophy (Fagan et al., 2009). Further, some studies showed that the CSF Aβ42/Aβ40 ratio is more clearly linked to AD than Aβ42 alone (Lewczuk et al., 2004; Wiltfang et al., 2007). The reason for the improved performance of Aβ42/Aβ40 ratio is still unclear, but it is hypothesized that Aβ40 can serve as an indicator of total Aβ and the ratio offsets the individual differences of total Aβ level in CSF (Lewczuk et al., 2015).

Besides measuring Aβ directly from CSF, positron emission tomography (PET) has been used to detect areas in which amyloid deposition is accumulated in the brain at early stages of AD (Blennow & Zetterberg, 2018). Both the Food and Drug Administration (FDA) and European Medicines Agency (EMA) have approved this method to rule out AD as the etiology of mild cognitive impairment (MCI) (Ritchie et al., 2017). For this purpose, an agent named Pittsburgh compound‐B ([11C]‐PIB) is used as ligand in PET imaging to indicate the amyloid deposition in the brain by inferring from the distribution area of ligand retention (Klunk et al., 2004). Apart from amyloid, tau agents are also used with PET imaging in numerous clinical trials (Brosch et al., 2017). Besides that, fluorodeoxyglucose (FDG) PET is used to detect decreased brain metabolism, which indicates the magnitude of neurodegeneration (Gray et al., 2012; Padilla et al., 2012; Teipel et al., 2015).

Taken together, on a micro perspective, the neurobiological, pathological progression of AD is accompanied by the occurrence of tau, and/or isoforms of the Aβ protein in the brain. Early diagnostic markers of AD either try to assess the concentration at which these pathological neurobiological (by)products are present in the brain or try to assess their distribution within the brain. However, CSF biomarkers are collected through lumbar puncture and the cost of PET testing is expensive. While these “micro” biomarkers of AD are currently the most sensitive, their costly methodologies and related high patient burden hinder the implementation of these practices in routine clinical examinations. As such, identifying less costly and burdensome alternatives could be beneficial for clinical practice.

## 3 EARLY DIAGNOSTIC MARKERS OF AD BASED ON PATHOLOGY ON THE MACRO LEVEL

We can look into the pathological progression of AD from both macro and micro perspectives. On the micro neurobiological level, Aβ and/or tau accumulation damages the neurons day by day. On the macro level, these pathological changes cause atrophy of certain brain structures, which can be detected by neuroimaging techniques such as magnetic resonance imaging (MRI).

The medial temporal lobe (MTL; including the hippocampus and its adjacent parahippocampal cortex, and the temporopolar cortex) is the brain region where the neuropathology of AD emerges and develops dramatically (Braak & Braak, 1991; Hyman et al., 1984). Braak and Braak demonstrated typical stages in which AD spreads through the brain. Earliest neurodegeneration seems to always occur in the entorhinal cortex (part of the parahippocampal cortex), and spread from there to the hippocampus. According to Braak stage theory (Stages I and II: transentorhinal region is affected with mild involvement of hippocampus; stages III and IV: both the entorhinal and transentorhinal regions are conspicuously affected with mild‐to‐moderate hippocampal and a low isocortical involvement; stages V and VI: the hippocampus is infested with NFTs and all isocortical association areas are severely affected), NFTs start to aggregate in the hippocampus from stage II onward and progressively damage it. From there, the disease spreads to the entire temporal lobe, and eventually reaches the isocortex at stage VI (Braak & Braak, 1991). It is not actually clear why the disease seems to follow these distinct stages, but it might have to do with the central role the MTL plays in contextualization, and memory consolidation, reconsolidation, and retrieval, making it one of the metabolically most active areas in the CNS.

Microscopic injuries chronically give rise to macroscopic atrophy that can then be visualized by in vivo techniques such as MRI. In the revised edition of NINCDS–ADRDA criteria (Dubois et al., 2007), MTL atrophy is proposed as a crucially supportive diagnostic criteria for AD in addition to clinical symptoms. Because of the relatively clear delineation of hippocampus anatomy, hippocampal volume (HV) estimated from MRI is considered a better structural biomarker compared to total MTL volume or entorhinal cortex volume. The European Federation of the Neurological Societies (EFNS) (Hort et al., 2010), the EMA (Hill et al., 2014), the National Institute on Aging and the Alzheimer's Association (NIAAA) (Albert et al., 2011), and the International Working Group (IWG) (Dubois et al., 2014) recommended HV as a supplementary biomarker indicating neuronal damage, and facilitating the clinical diagnosis of AD. Moreover, studies have linked hippocampal degeneration to CSF biomarkers of AD. For example, a smaller HV is positively correlated with decreased Aβ42 (Fagan et al., 2009) and increased amyloid and tau (Aschenbrenner et al., 2018). HV has further been found to be smaller in AD patients, and is summarized to be the best brain structure to discriminate AD patients from healthy controls (Jack et al., 1997). A bigger hippocampus is usually associated with better cognitive function (Ezzati et al., 2016; Hardcastle et al., 2020; Konishi et al., 2017; O'Shea et al., 2016) and lower risk of developing dementia (Mungas et al., 2002; Tabata et al., 2020). An autopsy study showed significantly larger HV in patients not cognitively impaired within 1 year of their death (Erten‐Lyons et al., 2009). Further, old patients with large hippocampi show preserved cognitive function despite pathological deterioration in the brain (Fotuhi et al., 2012). Taken together, these results confirm the notion that HV assessment can support the diagnosis of AD in the presence of clinical symptoms.

Since the concept of MCI was introduced in the 1990s (Flicker et al., 1991; Petersen et al., 1999; Petersen et al., 1995), much research has taken place to determine whether it is a preclinical stage of AD. MCI describes symptoms of slight cognitive dysfunction; yet people suffering from MCI do not fulfill the criteria of AD. However, compared to healthy controls, people with MCI are more likely to develop AD, with a conversion rate from 35% to 50.5% within 3 years (Luis et al., 2004; Palmer et al., 2002). It has further been suggested that the conversion rate within 1 year lies above 10% (Petersen et al., 2001), although a recent meta‐analysis argued that the conversion rate might be less than 10% (Mitchell & Shiri‐Feshki, 2009). On the transition from normal cognitive functioning to AD, MCI is a critical stage to focus on for the purpose of slowing down neurodegenerative progression, either by behavioral interventions or drug administration. Not everyone diagnosed with MCI goes on to develop AD, however, and not everyone developing AD went through a stage of MCI. Many other factors can cause MCI, including metabolic factors, vascular factors, dehydration, and inflammation. If the cause is reversible, the stage of MCI can then also revert to normal again. Recently, the term “amnestic mild cognitive impairment” has been introduced as it appears that this form of MCI, with a focus on cognitive impairment in the memory domain, is more strongly connected to subsequent development of AD. This would make sense in the light of the critical role both entorhinal cortex and hippocampus play in memory function.

Clearly, having MRI biomarkers that are sensitive at the preclinical stage (perhaps even pre‐MCI) would be beneficial to aid in the identification of those at risk. Unfortunately, the existing MRI biomarkers all fail to be sensitive enough for this purpose. On the transition from MCI to clinical AD, structural markers, which directly reflect neurodegeneration, have closer relationships with clinical symptoms than CSF biomarkers (like amyloid deposition) (Jack et al., 2009). Several longitudinal studies could demonstrate that decreased HV is positively related to increased risk of conversion from MCI to AD (Apostolova et al., 2006; Chupin et al., 2009; Eckerstrom et al., 2008; Fang et al., 2019; Jack et al., 1999; Risacher et al., 2009; Tabata, 2020). A meta‐analysis including 27 studies also supports the notion that HV is a good predictor of MCI‐to‐AD conversion (Hill et al., 2014). Overall, HV seems to be a good predictor of AD, at least at the MCI stage of the disease.

Since the pathological changes related to AD induce brain atrophy, changes in HV should be more informative compared to HV alone. Hippocampal atrophy through repeated assessment in longitudinal MRI designs is a marker closely associated with HV and harbors the advantage of a dynamic feature reflecting the progression of change. One previous study showed that combining two hippocampal metrics (HV and hippocampal atrophy) enhances the prediction of AD progression in preclinical individuals (McRae‐McKee et al., 2019). In accordance with the disease progression, significantly higher rates of hippocampal loss were found in AD and MCI patients compared to healthy controls (Fang et al., 2019; Jack et al., 2008; Ridha et al., 2006; Schuff et al., 2009). In harmony with HV, accelerated hippocampal atrophy also predicts a high possibility of conversion from MCI to AD (Henneman et al., 2009; Jack et al., 2004; McRae‐McKee et al., 2019; Vemuri et al., 2009). Several studies argue that hippocampal atrophy is the direct cause of (Mormino et al., 2009) or at least coupled to (Evans et al., 2018; Jack et al., 2004; Schuff et al., 2009; Thompson et al., 2004) cognitive decline, especially memory impairment. A clear disadvantage is, however, the need for repeated assessments sufficiently spaced in time to allow accurate assessment of atrophy.

A possible alternative biomarker is the assessment of adjacent hippocampal ventricular space in combination with the assessment of the gray matter volume. It can be argued that with HV loss, surrounding CSF space increases accordingly (Schoemaker et al., 2019). Previous studies have shown that ventricular expansion can correctly indicate presence of AD and MCI (Apostolova et al., 2013; Bartos et al., 2019; Coutu et al., 2016). Some studies even reported a better performance in measuring AD progress by assessing ventricular expansion compared with hippocampal atrophy (Macdonald et al., 2013; Thompson et al., 2004).

Overall, HV, the atrophy of the hippocampus, and the expansion of adjacent CSF space should be highly correlated with each other. These structural markers could thus be considered valid markers of MCI, AD, and their progression (Frisoni et al., 2010). Apart from the noninvasive and economic features of these measures, which is a clear advantage compared with CSF‐based markers of AD, MRI biomarkers present good anatomical features of degenerated regions, and the longitudinal assessment of MR images can display the dynamics of disease progression straightforwardly. As noted above, relying on a longitudinal assessment, diagnoses cannot be made promptly and the process of targeted intervention is delayed, which questions the applicability of these measures in the clinical context.

These limitations raise the question of whether there is a comprehensive biomarker embracing the features and information of the three markers together (HV, hippocampal atrophy, and ventricular expansion) while at the same time avoiding repeated measurements.

## 4 THE RATIO BETWEEN GRAY MATTER STRUCTURES AND DIRECTLY ADJACENT VENTRICULAR VOLUMES AS SUPERIOR DIAGNOSTIC MARKER OF BEGINNING NEURODEGENERATION?

Normally, HV decreases with aging, with the decline accelerating through the pathology of AD (Jack et al., 1997; Pruessner et al., 2001). However, a variety of factors also affect HV and cause variations among individuals, decreasing the diagnostic validity of HV on AD. First, different individuals have inherent variations in their hippocampal sizes. Initial hippocampal development early in life is typically independent of neuropathology, and related to a variety of different factors—genetic influences, availability of nutritional resources, amount of cognitive stimulation, and so forth. It can, however, be speculated that a larger generic HV might serve as a protective factor against neurodegeneration in old age, potentially providing a cognitive reserve. Be that as it may, there is significant variation of HV in young adulthood across the general population independent of disease (Lupien et al., 2007). Second, there are also some developmental factors other than aging that affect HV in middle to old adulthood (Schoemaker et al., 2019), for example, obesity (Jagust et al., 2005), environmental effects (Sullivan et al., 2001; Taylor et al., 2020), stress (Mirescu et al., 2004; Pruessner et al., 2005), chronic alcohol abuse (Agartz et al., 1999; Nixon et al., 2010), nutrition (Zainuddin & Thuret, 2012), and head trauma (Ariza et al., 2006; Beauchamp et al., 2011). However, HV in cross‐sectional studies merely indicates the current status without incorporating inherent and developmental factors causing additional variation, as mentioned above. As a result, studies investigating the association between HV and cognitive function show only moderate associations. On the one hand, a series of studies reported a positive correlation between HV and memory function (Ezzati et al., 2016; Hardcastle et al., 2020; Konishi et al., 2017; O'Shea et al., 2016); on the other hand, a meta‐analysis of 33 studies found little support for the “bigger‐is‐better” hypothesis, while the relationship between HV and memory is positive but weak in old people (Van Petten, 2004). We believe that a lot of this has to do with the inability of pure HV to differentiate the many factors contributing to the size of the structure at a given point in time.

A promising biomarker that can possibly overcome the limitations of the established structural markers associated with HV and hippocampal atrophy is the volumetric ratio of the hippocampus and its surrounding ventricle, abbreviated as hippocampal‐to‐ventricle ratio (Schoemaker et al., 2019). We believe there are clear advantages of HVR compared to HV. First, HVR takes into account information about HV and relates it to surrounding ventricular enlargement. Thus, HVR presents a dynamic feature that to some extent provides information that is normally available only through longitudinal data (i.e., a marker of change over time). As such, it could possibly provide information about the progress of HV loss, even though it is established cross‐sectionally. By providing a ratio, variations in hippocampal size that might be unrelated to volume loss caused by neurodegenerative factors are automatically controlled for.

There is first evidence that supports the view that HVR might be a superior measure to predict AD compared to HV alone. Bartos et al. (2019) showed that the ratio of hippocampus and the inferior part of the lateral ventricle allowed better discrimination of AD and control groups than absolute HV. This result partially provides the preliminary evidence of the superiority of HVR compared to HV. We expanded on this by taking the entire surrounding ventricle mass into account, and articulated the core rationale of the HVR (Coutu et al., 2016):

“To compute the HVR, we were guided by the assumption that the ventricle space directly surrounding the hippocampus increases as a function of atrophy or neurodegeneration of the hippocampus proper. Calculating a ratio combining a structural estimation of the target structure together with the ventricle space surrounding the target structure could provide an integrity index, which will indicate the preservation of the given structure (Schoemaker et al., 2019, p. 116108).”

In this paper, the validation of the HVR was first performed in a preclinical sample: both age and memory showed stronger negative correlations with HVR than compared to HV alone. Thus, the usefulness of HRV needs next to be confirmed in clinical studies that aim to discriminate AD from MCI and controls. As HVR assessment comes at low structural and economic costs, and low burden for the patient, its application in clinical practice would be highly feasible in the course of routine assessments.

**FIGURE 1:**
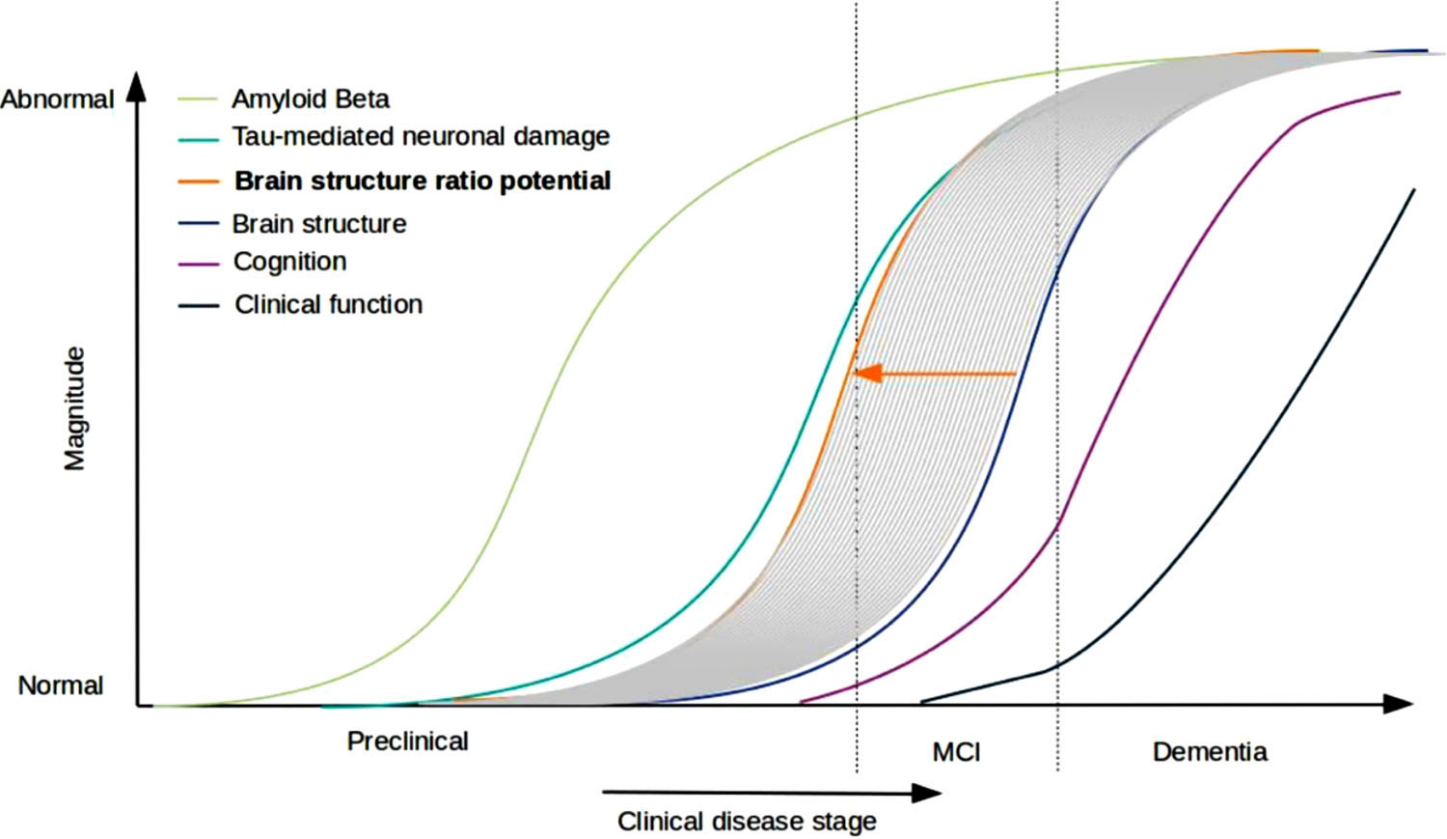
Schematic of how brain structure ratio might increase sensitivity for early diagnosis, based on Sperling et al. (2011) and Jack et al. (2010).

Taken together, we argue that the pathological decline of HV and surrounding structures often occur in MCI and AD. However, pure volume assessment of these brain structures is not sufficient to predict AD progression accurately. From preliminary results, HVR is introduced as potentially better index to represent structural integrity of the hippocampus. Thus, the consistent implementation of HVR in future studies might hold the potential to decrease the inconsistencies of results between studies. The possibly improved accuracy introduced by HVR would perhaps make it a better biomarker to predict MCI and prodromal AD in the preclinical period, which would be beneficial in clinical practice. Figure 1 is inspired by the illustration by Sperling et al. (2011) and Jack et al. (2010) depicting the sensitivity of different biomarkers of AD in predicting MCI and dementia at a preclinical stage. We suggest that a ratio of volume to ventricle marker has an improved sensitivity, which will shift the value of structural MRI to the left, into the proximity, and perhaps past some of the established wet marker for early diagnosis of AD.

A potential downside of HVR is the high cost in time and labor, as segmentation in the initial study was performed manually. This is a downside of all (manual) segmentation paradigms that the exact delineation of the individual anatomy takes considerable amounts of time. The segmentation includes the parcellation of two structures—the hippocampus and surrounding ventricle—consuming 1.5–2 h per brain for the expert rater. Therefore, automating the segmentation can largely reduce the overall time devoted to the segmentation. From pilot trials, we know that by using automated algorithms, for example, the Multiple Automatically Generated Templates (MAGeT) (Chakravarty et al., 2013; Pipitone et al., 2014) package, segmentation time can be reduced to around 30 min, only requiring quality control of the automated segmentation. Thus, for studies in large populations samples the implementation of the automated pipelines can largely enhance the practicability of utilizing the HVR index. If employed in individual subjects in the context of clinical assessments, this can be considered less of a concern though.

## 5 CONCLUSION

In this opinion paper, we first reviewed the current status of dementia and AD in the population and showed the necessity and importance for sensitive biomarkers already at the preclinical stage. Then we reviewed existing early diagnostic markers commonly used for AD: CSF biomarkers including Aβ42, T‐tau, and P‐tau; PET imaging of amyloid and tau; Pittsburgh compound‐B; and FDG PET. Next, we focused on a structural neuroimaging biomarker, HV, summarizing previous results. Here, we described the relationship between HV and MCI/AD as well as the predictive value of HV on MCI‐to‐AD conversion. Also, we presented two markers related to AD—hippocampal atrophy and ventricular expansion. At last, we put forward that ratios between structural volumes, for example, the HVR, might be promising diagnostic markers of AD. Finally, we elaborated the rationale of HVR and showed the evidence for preliminary validation of the HVR.

It remains to be seen whether HVR has the potential to increase the early diagnostic accuracy of hippocampal structural integrity in AD and MCI and decrease the discrepancy of research findings. Clearly, HVR needs to be applied to clinical studies with MCI and AD patients. Finally, the implementation of HVR in longitudinal studies investigating the possibility of conversion from normal to MCI/AD would allow validating its predictive power in direct comparison with longitudinal analyses.

In conclusion, we suggest that depicting the ratio between atrophied structures and direct adjacent regions that have occupied the atrophied region can be a more informative measure compared to absolute structural volumes of atrophied regions alone. Extending that argument, ratio measures of regions that are affected by AD even earlier than the hippocampus (i.e., the transentorhinal region) could even be more informative compared to the proposed HVR. Overall, we think that this line of research holds the promise of providing valuable clinical information, could help with the diagnosis of AD at an early preclinical stage, and could thus help in the prevention and intervention and the lowering of individual, as well as societal costs associated with dementia.

## CONFLICT OF INTEREST STATEMENT

The authors declare no conflicts of interest.

### PEER REVIEW

The peer review history for this article is available at https://publons.com/publon/10.1002/brb3.2925.

## Data Availability

Data sharing not applicable to this article as no datasets were generated or analyzed during the current study.
